# Living well with long-term conditions: A quantitative investigation into the influences of illness and healthcare experiences

**DOI:** 10.1177/17423953261417134

**Published:** 2026-01-27

**Authors:** Poppy Dilks, Isabelle Ball, Moitree Banerjee

**Affiliations:** Department of Psychology and Counselling, 2476 University of Chichester, Chichester, UK

**Keywords:** Long-term conditions, healthcare experience, illness experience, living well, quality of life

## Abstract

**Objectives:**

To examine whether illness or healthcare experiences have a more significant influence on living well, and which factors in these experiences have the most influence.

**Methods:**

Information collected included demographic data, illness and healthcare experience, and the LTCQ to measure living well. Data was collected via online survey platform Qualtrics. Two separate 2-stage hierarchical multiple regressions were run to investigate how much variance in living well with long-term conditions is accounted for by established and exploratory illness and healthcare experience factors.

**Results:**

70 participants met the inclusion criteria of the study, with 54 included in the analysis. Results showed that illness experience had a significant influence on living well while healthcare experience did not. The factors of illness intrusiveness in illness experience and patient assessment of chronic illness care in healthcare experience significantly impacted living well.

**Discussion:** This study examines the influences of illness and healthcare experiences on the ability to live well with LTCs. Future research could focus on specific LTCs and compare which factors they find significantly affect living well. The findings pave the way for future explorations into the factors influencing living well differ between LTCs and the best interventions to improve living well with LTCs.

Living with health conditions can prominently affect an individual's life, particularly if they have a long-term condition (LTC). In the United Kingdom, almost half of the population report having an LTC, which is a condition that cannot currently be cured but is controlled by medication/other treatment.^1,2^ LTCs affect quality of life by limiting activities and worsening mental well-being.^2,3^ Quality of life is an individual's perceptions of their position and feeling of overall satisfaction in their life. This concept was expanded on to include the impact that someone's ailment has on different aspects of their life, creating health-related quality of life (HR-QOL).^4^

Recently, HR-QOL has shifted to ‘living well’, particularly living well with an LTC.^5,6^ Potter et al. built on this by including not only traditional HR-QOL domains, but also treatment burden and confidence in self-management.^7^ When defining living well, the inclusion of patients’ perspectives allows for inclusive terminology and patient-centred care to be emphasised. Living well with an LTC is measured by the recently developed Long-Term Conditions Questionnaire (LTCQ).^7^ The holistic nature of this questionnaire makes it suitable to use in clinical settings, such as the National Health Service (NHS).

Research on factors impacting living well is in its infancy. While biomedical factors have been explored, emerging evidence suggests further multidimensional interpersonal and psychosocial variables can play a role.^5,6^ Therefore, the overarching themes of illness experience and healthcare experience were created to categorise these. Within these, some factors are already established with a multitude of research from a wide range of that emphasised their impact on HR-QOL, while exploratory factors had limited research, focusing on a single condition or area of QOL.^7^

Illness experience contains the established factors of illness intrusiveness, social/role activities limitations, and a negative perception of the illness. Illness intrusiveness is the perceived interference an LTC has on daily life, and has been associated with increased fatigue and worsened physical and mental HR-QOL.^8^ Further research also shows that having a social support network is positively associated with HR-QOL while the stronger the negative perceptions of the illness, the worse the HR-QOL.^9,10^ These factors represent aspects of the illness experience, that with further support in a community and healthcare setting, can be positively changed. For example, as the number of LTCs increases with age, older adults experience events such as retirement, death of a spouse, and children leaving the home, lessening their opportunities to participate in social settings.^10^ This is a relatively curable hindrance with additional community support programmes. Exploratory factors are energy balance behaviours and pain severity, both of which are subjective and under-researched.^11,12^ Research into health distress has been limited to specific conditions, finding that untreated diabetes distress correlates with impaired HR-QOL.^11–13^

Healthcare experience includes the established impactful factor of healthcare utilisation, while a lesser established exploratory factor is patient assessment of chronic illness.^14,15^ These are significantly more difficult to improve at a fundamental level but lay the foundation for any form of healthcare experience. When examining demographic factors, established variables include the impact of age and socio-economic status, contrasting age at diagnosis and duration/length of LTC, which are exploratory in nature.^16–18^

The present study will compare the effects of illness experiences and healthcare experiences on living well with LTCs to examine which factors have a more significant influence on living well. Findings will allow healthcare providers to prioritise exploring focused interventions to target these factors and consequently improve patients’ living well with their LTC.

## Methods

### Participants

Participants were recruited via social media, posters, and the survey exchange websites. Undergraduate students were recruited through research credit schemes. The study was approved in accordance with the Research Ethics Policy of the University of Chichester.

Of 129 participants who agreed to take part in the study, 70 fit the inclusion criteria of identifying as having a LTC not considered a disability (54%). Among those who fit the criteria, 16 participants did not complete the full questionnaire, resulting in 54 participants being included in the analysis (42% of the original sample). Participants confirmed they had an LTC that they did not define as a disability. Due to sensitivity and stigma around visible and invisible LTCs, participants were not required to reveal their condition.^19^ 27.8% of participants were students at the University of Chichester.

Of the analysed data, mean age was 37 years (*SD* = 14.85, range = 18–71), 80% were female and 99% were cisgender. Participants socio-economic status ranged from 0 (least money, little education, no job) and 9 (most money, highest amount of schooling, respected jobs) on the socio-economic ladder (*M* = 5.20, *SD* = 1.78). Participants age at diagnosis ranged from 1 to 60 (*M* = 24.65, *SD* = 14.45), while their duration of LTC ranged from 1 year to 54 years (*M* = 14.11, *SD* = 12.36). 55.7% of participants identified as heterosexual and 67.1% were white British [See Appendix 1].

### Materials

Demographic data included age, sex, gender, sexual orientation, ethnicity, socio-economic status, age at diagnosis of LTC, and duration of LTC [See Appendix 2].

#### The Adapted Illness Intrusiveness Scale

^20,21^ The Adapted Illness Intrusiveness Scale consists of participants indicating their level of agreement with the statement “How much does your illness(es) and/or its treatment interfere with:” for items such as “Your feeling of being healthy?”.

#### The Energy/Fatigue Scale

^22,23^ The Energy/Fatigue Scale consists of participants indicating their level of agreement with the statement “How much time during the past 4 weeks…” for items such as “Did you feel worn out?”.

#### The Pain Severity Scale

^22,23^ The Pain Severity Scale consists of participants rating the severity, frequency, and length of physical discomfort or pain.

#### The Health Distress Scale

^22,23^ The Health Distress Scale consists of participants indicating their level of agreement with the statement “How much time during the past month…” for items such as “Were you discouraged by your health problems?”.

#### The Social/Role Activities Limitations Scale

^23^ The Social/Role Activities Limitations Scale consists of participants indicating their level of agreement with the statement “During the past 4 weeks, how much…” for items such as “Has your health interfered with your hobbies or recreational activities?”.

#### The Brief Illness Perceptions Questionnaire

^24^ The Brief Illness Perceptions Questionnaire consists of participants choosing the number that best corresponded to their views.

#### The Healthcare Utilisation Scale

^23,25^ The Healthcare Utilisation Scale consists of participants indicating their answers with a number of visits/times/nights.

#### The Patient Assessment of Chronic Illness Care Scale

^26^ The Patient Assessment of Chronic Illness Care Scale consists of participants indicating their level of agreement with the statement “Over the past 6 months, when I received care for my chronic conditions, I was:” for items such as “Asked for my ideas when we made a treatment plan.”.

#### The Long-Term Conditions Questionnaire

^7^ The LTCQ consists of participants indicating their level of agreement with the statement “Please think about your long-term health condition(s) over the past four weeks. How often have you…” for items such as “Felt able to cope well with your health condition(s)?”.

### Procedure

Participants completed the survey using the platform Qualtrics.^27^ Participants read the information surrounding the research, their right to withdraw, and consented [See Appendix 3]. Participants answered a series of questionnaires and were fully debriefed. All participants were signposted to support for LTCs in and outside the university and given the contact details of the researcher.

### Data analysis

The independent variables were divided into two categories: illness experience and healthcare experience. Of these two categories, the variables were categorised as either an established factor in block one, or an exploratory factor in block two (see Table 1).

**Table 1.** Factors of illness and healthcare experience categorised into established and exploratory for the regression models.

Illness Experience		Healthcare Experience	
Established	Exploratory	Established	Exploratory
Illness Intrusiveness	Energy	Healthcare Utilisation	Patient Assessment of Chronic Illness Care
Social/Role Activities Limitations	Pain Severity	Age	Age at Diagnosis
Illness Perception	Health Distress	Socio-Economic Status	Length of LTC

#### Pearson's correlation coefficient

All statistical analyses were completed in SPSS.^28^ A Pearson's correlation coefficient was conducted to investigate the extent of association between variables and the degree of variation (see Tables 2 and 3).

**Table 2.** A correlation matrix for illness experience variables.

Factor	Mean (*SD*)	Living Well	Illness Intrusiveness	Energy	Pain Severity	Health Distress	Social/Role Activities Limitations	Illness Perception
Living Well	59.05 (16.58)	x						
Illness Intrusiveness	34.59 (12.10)	−.70**	x					
Energy	2.08 (.86)	.45**	−.45**	-				
Pain Severity	3.69 (1.32)	−.43**	.51**	−.55**	x			
Health Distress	3.26 (1.11)	−.53**	.60**	−.42**	.50**	x		
Social/Role Activities Limitations	2.56 (1.05)	−.61**	.77**	−.50**	.63**	.70**	x	
Illness Perception	47.65 (7.69)	−.29*	.45**	−.24*	.42**	.42**	.46**	x

*p < .05, **p < .001

d.f. = 54

**Table 3.** A correlation matrix for healthcare experiences variables.

Factor	Mean (*SD*)	Living Well	Healthcare Utilisation 1	Healthcare Utilisation 2	Healthcare Utilisation 3	Healthcare Utilisation 4	Patient Assessment of Chronic Illness Care	Age at diagnosis	Length of LTC	Age	Socio economic status
Living Well	59.27 (16.66)	x									
Healthcare Utilisation 1	2.72 (2.64)	−.23	x								
Healthcare Utilisation 2	.42 (1.01)	−.11	−.14	x							
Healthcare Utilisation 3	.11 (.58)	−.15	−.12	.74**	x						
Healthcare Utilisation 4	.11 (.51)	.01	−.11	.55**	.75**	x					
Patient Assessment of Chronic Illness Care	2.21 (.92)	.38*	−.02	.01	−.16	−.14	x				
Age at diagnosis	24.08 (14.44)	−.21	.37*	.02	.01	−.11	.03	x			
Length of LTC	15.11 (12.80)	.11	−.19	−0.5	−.10	−.08	−.14	−.46**	.x		
Age	36.62 (14.85)	−.13	.21	−.07	−.04	−.12	−.21	.58**	.36*	x	
Socio economic status	5.17 (1.76)	.06	.05	−.13	−.11	−.09	.20	.22	−.01	.12	x

*p < .05, **p < .001

d.f. = 53

#### Multiple hierarchical regression

Examining the assumptions concluded that the data met the assumptions for no multicollinearity, no independent errors, and no outliers [See Appendix 4]. Analysis of scatter plots demonstrated that the assumptions of linearity and homogeneity were satisfied. Therefore, the multiple hierarchical regressions were run (see Tables 4 and 5).

**Table 4.** A table providing a summary of the hierarchical regression analysis between the six illness experience predictor variables on levels of living well with LTCs.

	Model 1		Model 2	
Variable	*B*	*β*	*B*	*β*
Constant	87.90		80.68	
Illness intrusiveness	−.81	−.59	−.76	−.55
Social activities	−2.79	−.18	−.69	−.55
Illness perception	.11	.05	.14	.07
Energy			2.58	.13
Pain severity			−.06	−.01
Health distress			−2.10	−.14
*R^2^*	.51		.53	
*F*	17.02		8.86	
*ΔR^2^*	.51		.03	
*ΔF*	17.02		.85	

**Table 5.** A table providing a summary of the hierarchical regression analysis between the nine healthcare experience predictor variables on levels of living well with LTCs.

	Model 1		Model 2	
Variable	*B*	*β*	*B*	*β*
Constant	63.53		47.03	
Healthcare Utilisation 1	−1.46	−.23	−1.18	−.19
Healthcare Utilisation 2	−.049	−.00	−1.65	−.10
Healthcare Utilisation 3	−10.69	−.37	−6.57	−.23
Healthcare Utilisation 4	8.64	.26	8.77	.27
Age	−.08	−.07	.12	.11
Socio economic status	.58	.06	.00	.00
PACIC			7.35	.41
Age at diagnosis			−.20	−.18
Length of LTC			.01	.01
*R^2^*	.13		.27	
*F*	1.11		1.77	
*ΔR^2^*	.13		.14	
*ΔF*	1.11		2.81	

## Results

### Regression one: illness experiences

Pearson's correlations were computed for each variable. Table 2 demonstrates the correlation matrix. A strong positive correlation between illness intrusiveness and social/role activities limitations was observed. Additionally, a strong negative correlation can be observed between living well and illness intrusiveness.

#### Hierarchical regression analysis

To investigate the extent to which illness experience predicted levels of living well with LTCs, a 2-stage hierarchical multiple regression was conducted (Table 4).

The hierarchical multiple regression revealed that at stage 1, illness intrusiveness, social / role activities limitations, and illness perceptions contributed significantly to the regression model, (*F*(3, 50) = 17.02, *p* < .001). The relationship between variables were strong (*R* = .71) and accounted for approximately 51% (*ΔR^2^* = .51) of the variance in living well scores. While illness intrusiveness had a statistically significant impact (*β* = −.59, *t*(54) = −3.73, *p* < .001), both social/role activities limitations and illness perceptions did not. Adding stage 2 to the regression model accounted for an additional 3% *(ΔR^2^* = .03) of variation in living well scores and this change in *R^2^* was insignificant, (*F*(3, 47) = .85, *p* = .473) but the relationship between the variables were strong (*R* = .73). Illness intrusiveness continued to have a significant impact on living well (*β* = −.55, *t*(54) = −3.44, *p* < .001), and none of the additional stage 2 factors of energy, pain severity, health distress or the stage 1 factors of social/role activities limitations and illness perceptions influenced living well.

The full model of illness intrusiveness, social activities, illness perceptions, energy, pain severity, and health distress to predict levels of living well with LTCs was statistically significant, *R^2^* = .53, *F*(6, 47) = 8.86, *p* < .001, *ΔR^2^* = .03.

### Regression two: healthcare experiences

#### Pearson's correlation coefficient

Pearson's correlations were computed for each variable. Table 3 demonstrates the correlation matrix. There was a strong positive correlation between current age and age at diagnosis. Additionally, a strong negative correlation can be observed between age at diagnosis and length of LTC.

#### Hierarchical regression analysis

To investigate the extent to which healthcare experience predicted levels of living well with LTCs, a 2-stage hierarchical multiple regression was conducted.

The hierarchical multiple regression revealed that at stage 1, healthcare utilisation, age, and socio-economic status contributed insignificantly to the regression model, (*F*(6, 46) = 1.11, *p* = .370). The relationship between variables was weak (*R* = .36) and accounted for approximately 13% (*ΔR^2^* = .13) of the variance in living well scores. Neither healthcare utilisation, age, or socio-economic status in stage 1 had a statistically significant impact.

Adding stage 2 to the regression model accounted for an additional 14% *(ΔR^2^* = .14) of variation in living well scores and this change in *R^2^* was insignificant, (*F*(3, 43) = 2.81, *p* = .051). The relationship between the variables was moderately strong (*R* = .52).

Patient assessment of chronic illness care (PACIC) had a significant impact on living well at stage 2 (*β* = .41, *t*(53) = −3.44, *p* = .007), while none of the additional stage 2 factors of age at diagnosis or length of LTC, or the stage 1 factors of healthcare utilisation, age, or socio-economic influenced living well.

The full model of healthcare utilisation, age, socio-economic status, PACIC, age at diagnosis, and duration of LTC to predict levels of living well with LTCs was statistically insignificant, *R^2^* = .52, *F*(3, 43) = 1.77, *p* = .103, *ΔR^2^* = .14.

## Discussion

The study aimed to examine whether illness or healthcare experiences have a more significant impact on living well LTCs, and which factors in these experiences have the most influence. The full models show that illness experience significantly predicts living well with LTCs, while healthcare experience does not. These findings are consistent with the suggestion that illness experience is subjective and can have a significant impact on both the person experiencing it and the people around them through negatively impacting HR-QOL.^4,29^ The experience of LTCs has been established to negatively impact patients’ assumptions of the world, the future, and the self. This can result in depleted capacity to continue living their everyday lives, and potentially increase hospital utilisation.^30^ These results emphasise the need for psychologists and mental health support services to be involved more routinely in LTC clinics, as they have been proven to lessen time in hospital.^15^ However, this may prove difficult due to the current shortage of psychologists within the National Health Service.^31^

### Summary of main findings

The finding that healthcare experience was not a significant predictor of living well presents a challenge to previous research. The effect of healthcare experiences on quality of life has been emphasised in multiple studies, finding that the worse a person's experience of healthcare service, the worse their quality of life.^32,33^ A potential explanation for the contradiction is that this study, while not explicitly, likely included participants with diverse health conditions, potentially encompassing those with less severe conditions who may require infrequent medical consultations.

This is unlike most LTC studies which focus on one severe LTC, such as Alessy et al.'s finding that a late-stage cancer diagnosis was consistently associated with poorer cancer care experience compared to an early-stage diagnosis.^34^ Therefore, caution is required when generalising this study's findings, as individuals with different illnesses may attribute varying levels of importance to factors influencing living well. While healthcare providers should focus on illness experience interventions with patients struggling to live well with their LTC, healthcare experience factors should be considered as well.

**Figure 1.**
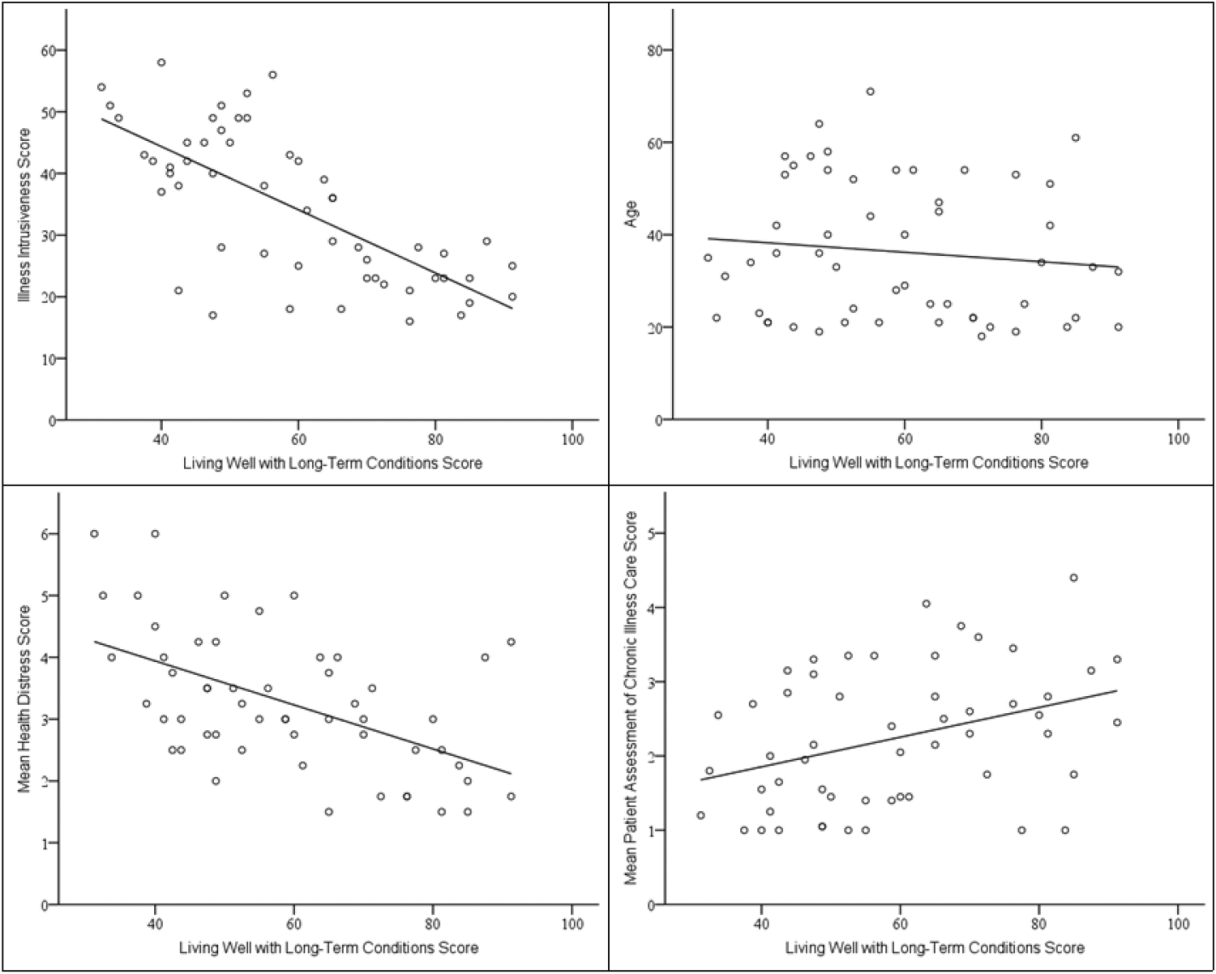
Scatter plots with regression lines for living well with long-term conditions and illness intrusiveness scores (top left), health distress scores (bottom left), age (top right), and patient assessment of chronic care scores (bottom right).

Illness intrusiveness had a statistically significant impact on living well with LTCs, in both stages (Figure 1). This supports previous research findings that illness intrusiveness is a strong predictor of HR-QOL and can be very detrimental, disrupting their lifestyle, activities, and interests, threatening psychological wellbeing.^35^ The furthering of emotional distress in this way has shown to impair quality of life, highlighting the need for self-management to be supported by healthcare teams.^8^ When adding the exploratory variables at stage 2, the explanatory power increased by 2%, indicating that energy, pain severity, and health distress are weak predictors. Further research around these factors is needed to clarify their impact on HR-QOL. The second regression revealed that at stage 1, the established healthcare experience variables accounted for approximately 13% of the variance in living well scores. None of the established variables of healthcare utilisation, age, and socio-economic status had a statistically significant impact on living well with LTCs, at stage 1 or 2. This contradicts previous research, possibly explained by the participant sample used in the current study. With nearly one-third of the participants being students, they may have a low health utilisation score due to the possibly mild nature of their condition. When adding the exploratory variables at stage 2, the explanatory power increased by 14%, indicating that they are moderately weak predictors. PACIC significantly impacted living well with LTCs. This supports evidence that in an expected range of illnesses, satisfaction with care correlates with quality of life.^36,37^ These results stress the need for healthcare managers to understand the aspects that improve patient care and find ways to support the healthcare providers in continuing these.

### Strengths and limitations

The current study participants did not specify their condition, allowing the results to be applied to a wide variety of people. The results allow for healthcare providers to prioritise supporting patients with the impact of their illness on their usual activities, and advocate for any issues they have with their care. However, specific conditions may benefit from further investigation. Future research could focus on most prevalent LTCs and compare with current findings.

One limitation of the study is that LTCQ uses a self-report measure, which have been criticised as causing possible biased answers and resulting in loss of construct validity.^38^ However, research highlights the strength of self-report measures, as the extremely personal and individualistic nature of LTCs and their consequences may not be represented by observation from others.^39,40^ The current study utilised Potter et al.'s LTCQ to measure living well with LTCs.^7^ The LTCQ is widely applicable to a range of LTCs and provides a reliable, valid, and holistic measure of living well with LTCs.^7^ However, LTCQ has been criticised for lacking clarity on how healthcare providers move forward with the results, such as implementing care pathways or referral processes.^6^ The Living with Long-Term Conditions Scale has recently been developed by Ambrosio et al., to tackle these issues.^5,6^ Therefore, future research could replicate the present study using the Living with Long-Term Conditions Scale.

### Conclusion

This study used two multiple hierarchical regressions to evaluate the influences of illness and healthcare experiences on the ability to live well with LTCs. Illness experience had a significant impact on living well with LTCs. The established factor of illness intrusiveness and the exploratory factor of PACIC significantly impacted living well. By prioritising patient perspectives in the concept of living well, the holistic nature of patient centred care is enhanced. These findings pave the way for future research to explore if factors influencing living well differ between LTCs and find the best interventions to improve living well with LTCs.

## Appendices

### Appendix 1

#### Demographic frequency table

Frequency and percent of answers on the Socio-Economic Scale, sexual orientation, and ethnicity or ethnic group.[Table table6-17423953261417134]

**Table table6-17423953261417134:**

	Variable	Frequency	Percent
Socio-economic Status (Number on the Ladder)	0	1	1.4
	1	1	1.4
	2	3	4.3
	3	4	5.7
	4	11	15.7
	5	17	24.3
	6	13	18.6
	7	9	12.9
	8	5	7.1
	9	1	1.4
Sexual Orientation	Bisexual	11	15.7
	Gay or Lesbian	7	10
	Heterosexual or Straight	39	55.7
	Other sexual orientation	3	4.3
	Prefer not to say	5	7.1
Ethnicity or Ethnic Group	Arab	1	1.4
	Asian Chinese	1	1.4
	Asian Pakistani	1	1.4
	Any other Asian background	1	1.4
	Black African	2	2.9
	Black Caribbean	1	1.4
	White British	47	67.1
	White Irish	1	1.4
	White Roma	1	1.4
	Any other White background	6	8.6
	Not known	1	1.4
	Prefer Not To Say	2	2.9

### Appendix 2

#### Scale information

The names, items, scale anchors, possible score range, scoring system, and Cronbach's alpha's of each scale used.[Table table7-17423953261417134]

**Table table7-17423953261417134:**

Scale Name	Items	Scale Anchor	Possible Score Range	Scoring	Given α	Current Study α	α revisions
The Adapted Illness Intrusiveness Scale	13 items	7-point Likert scale ranging from “Not very much” to “Very much”	13–91	A higher score indicates more intrusion	.89	.90	n/a
The Energy/Fatigue Scale	5 items	6-point Likert scale ranging from “None of the time” to “All of the time”	0–25	A higher score indicates more energy (items 1 and 3 were reverse scored)	.89	.81	n/a
The Pain Severity Scale	5 items	The level of agreement on the 10-point scale ranged from “None” to “As bad as you can imagine” The level of agreement on the 6-point scale ranged from “Never” to “Every day or almost every day”; “None” to “Very severe”; “Didn’t have any” to “More than 2 days”	5–38	A higher score indicates more pain	.88	.87	n/a
The Health Distress Scale	4 items	6-point Likert scale ranging from “None of the time” to “All of the time”	0–20	A higher score indicates more distress	.87	.89	n/a
The Social/Role Activities Limitations Scale	4 items	5-point Likert scale ranging from “Not at all” to “Almost totally”	0–16	A higher score indicates greater activities limitations	.91	.92	n/a
The Brief Illness Perceptions Questionnaire	10 items	11-point scale with different ranges; these statements ranged from “Absolutely no control” to “Extreme amount of control” and “Not concerned at all” to “Extremely concerned”	0–80	A higher score indicates a more threatening perception of the illness	.69	.86	Cronbach's alpha was .16, so questions 2, 3, 4 and 7 were removed.
The Healthcare Utilisation Scale	4 items	The participants indicated their answers with a number of visits/times/nights	0-	A higher score indicates more healthcare utilisation	-	.85	Cronbach's alpha was .07, so question 1 was removed.
The Patient Assessment Of Chronic Illness Care Scale	20 items	5-point Likert scale ranging from “None of the time” to “Always”	20–100	A higher score indicates better care	.93	.94	n/a
The Long-Term Conditions Questionnaire	20 items	5-point Likert scale ranging from “Never” to “Always”	20–100	A higher score indicates a higher quality of life/level of living well with LTCs (items 9 to 15 were reversed scored)	.95	.88	n/a

### Appendix 3

#### Extract from the participant information sheet

*Why is the study being conducted?*

We would like to invite you to take part in our research study. Before you decide it is important that you understand why we are doing the research and what we are asking of you. Please read this information. If you have questions, or if you would like more information, please ask us.

Who is carrying out the research? This research project is being undertaken as part of an Undergraduate study for Poppy Dilks.

Why is the research being done? The purpose of this project is to investigate the influences of illness and healthcare experience on living well with a long-term condition.

Why are you invited? You are invited to participate in this research project because you have a long-term condition.

We will now answer some important questions about this research.

*What does participation involve?*

Your participation will involve a questionnaire that will take approximately 15 min of your time.

*Questions will include:*

How much time during the past month were you discouraged by your health problems?

How much control do you feel you have over your illness?

*What happens if you change your mind and want to withdraw?*

Your participation in this research project is entirely voluntary. If you do agree to participate you can withdraw from the research project without comment or penalty. You can withdraw anytime during the questionnaire. If you withdraw within 2 weeks after your questionnaire, on request any information already obtained that can be linked to you will be destroyed. If you wish to exercise your right to withdraw consent or request erasure of personal information after 2 weeks, it may not be possible to erase your data without seriously impairing the achievement of the research objectives and therefore we may not be able to accommodate this request. Your decision to participate or not participate will in no way impact upon your current or future relationship with the University (for example your grades) or [associated external organisation].

*What are the possible benefits for me if I take part?*

To recognise your contribution, should you choose to participate, the research team is offering 15 min of participation credit to psychology students at the University of Chichester.

*What are the possible risks for me if I take part?*

There are minimal risks associated with your participation in this research project. These include concerns about your condition and experiences. These will be minimised by signposting to support at the end of the study.

### Appendix 4

#### Collinearity tests table

Collinearity tests of the factors.[Table table8-17423953261417134]

**Table table8-17423953261417134:**

Factor	Tolerance	VIF
Illness Intrusiveness	.39	2.58
Social / Role Activities Limitations	.28	3.58
Illness Perceptions	.73	1.37
Energy	.65	1.55
Pain Severity	.52	1.94
Health Distress	.49	2.05
Healthcare Utilisation 1	.84	1.20
Healthcare Utilisation 2	.40	2.53
Healthcare Utilisation 3	.27	3.76
Healthcare Utilisation 4	.42	2.40
Age	.15	6.51
Socio-Economic Status	.85	1.18
Patient Assessment of Chronic Illness Care	.82	1.18
Age at Diagnosis	.13	7.60
Duration of LTC	.19	5.41
